# Supplementary material for: Limited Differences in Insect Herbivory on Young White Spruce Growing in Small Open Plantations and under Natural Canopies in Boreal Mixed Forests
Source: Insects. 2024 Mar 15;15(3):196. doi: 10.3390/insects15030196 (PMC10971650; doi:10.3390/insects15030196)
Supplement: Supplementary file 1 [file insects-15-00196-s001.zip › Supplementary data damage paper.pdf]

Supplementary data

Table S1. Environmental parameters and plant traits were compared between habitats in late summer 2020 and 2021 via t-test analysis.

Habitat	Forest			Plantation					
Variables	N	Mean	SD	N	Mean	SD	*Df*	*p-value*	*Significance*
Tree height (m)	183	3.02	0.644	200	2.96	0.507	380.38	0.919	ns
Developed buds	176	24.6	12.33	189	31.6	15.19	360.39	<0.0001	***
Soil Temp (ºC)	180	21.50	4.15	200	23.46	4.95	368.88	<0.0001	***
Soil Humidity (%)	181	13.32	8.47	200	19.28	10.74	374.18	<0.002	***
Canopy cover (%)	183	87.43	19.34	200	37.07	23.49	375.83	<0.0001	***
Lateral growth 2021 (cm)	91	5.9	1.89	100	10.17	3.46	187.86	<0.0001	***
Lateral growth 2020 (cm)	134	7.41	3	200	13.61	4.38	331.53	<0.0001	***
Lateral growth 2019 (cm)	134	7.36	2.56	200	13.2	3.9	332.39	<0.0001	***
Lateral growth 2018 (cm)	89	8.29	3.12	100	13.83	3.06	187.71	<0.0001	***
Needle Toughness (gm)	80	50.02	11.76	77	56.4	20.6	152.69	0.02	**

*(c)Statistical significance markers: ns= not significant; * p<0.01 ** p<0.05; ***p<0.001*

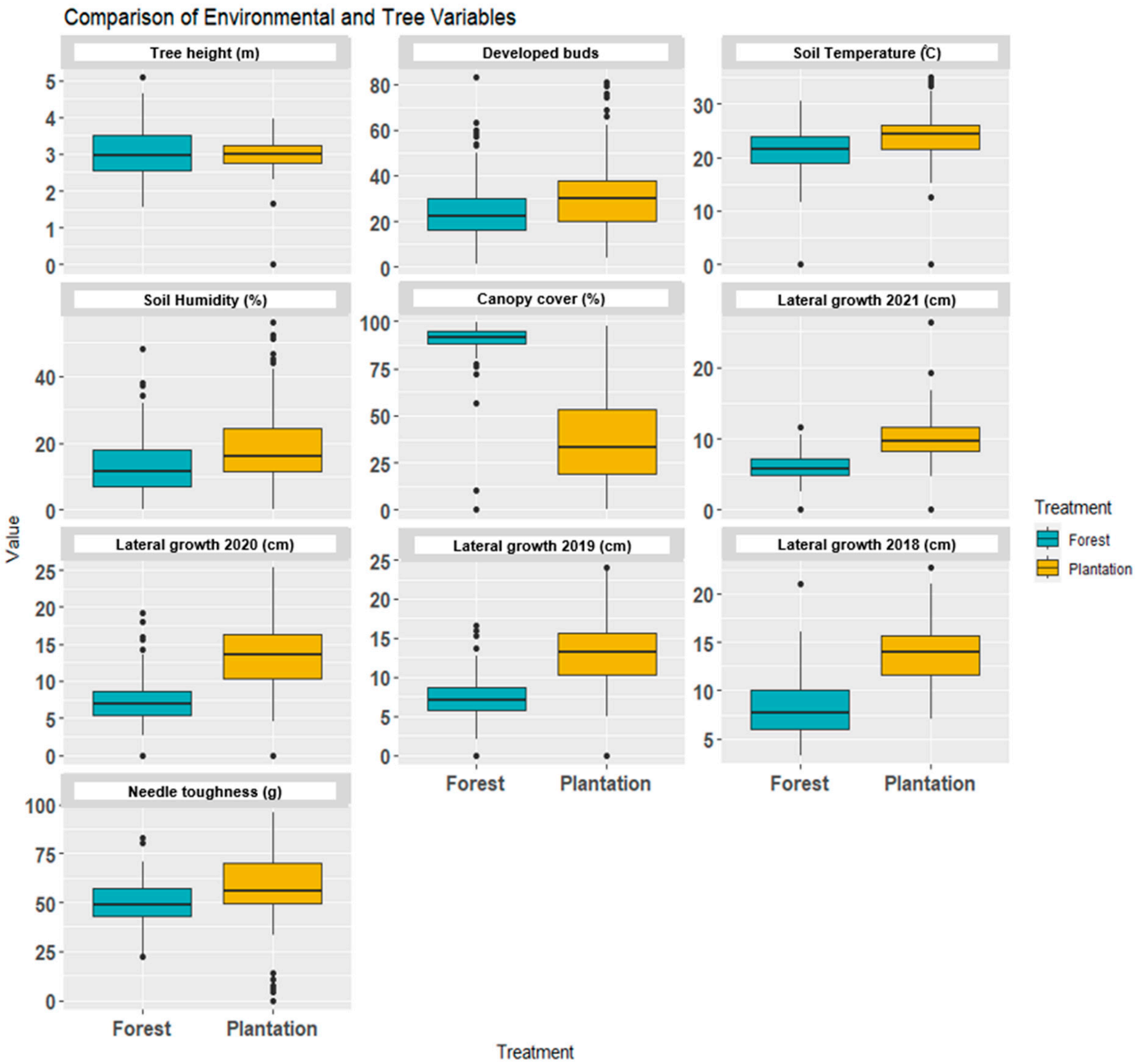

Figure S1. Graphical illustrations of ecological parameter comparisons between two habitats employing t-tests and statistical significance were determined (*p < 0.05, **p < 0.01, ***p < 0.001). The boxplots visually summarize central tendencies, spreads, and potential differences between treatments. Subplots feature key parameters, with treatment on the x-axis and corresponding measurement values on the y-axis. Variables include Tree height (m), Developed buds, Soil temperature (℃), Soil humidity (%), Canopy cover (%), Lateral shoot growth rate (cm) from 2018 to 2021, and Needle toughness (g).

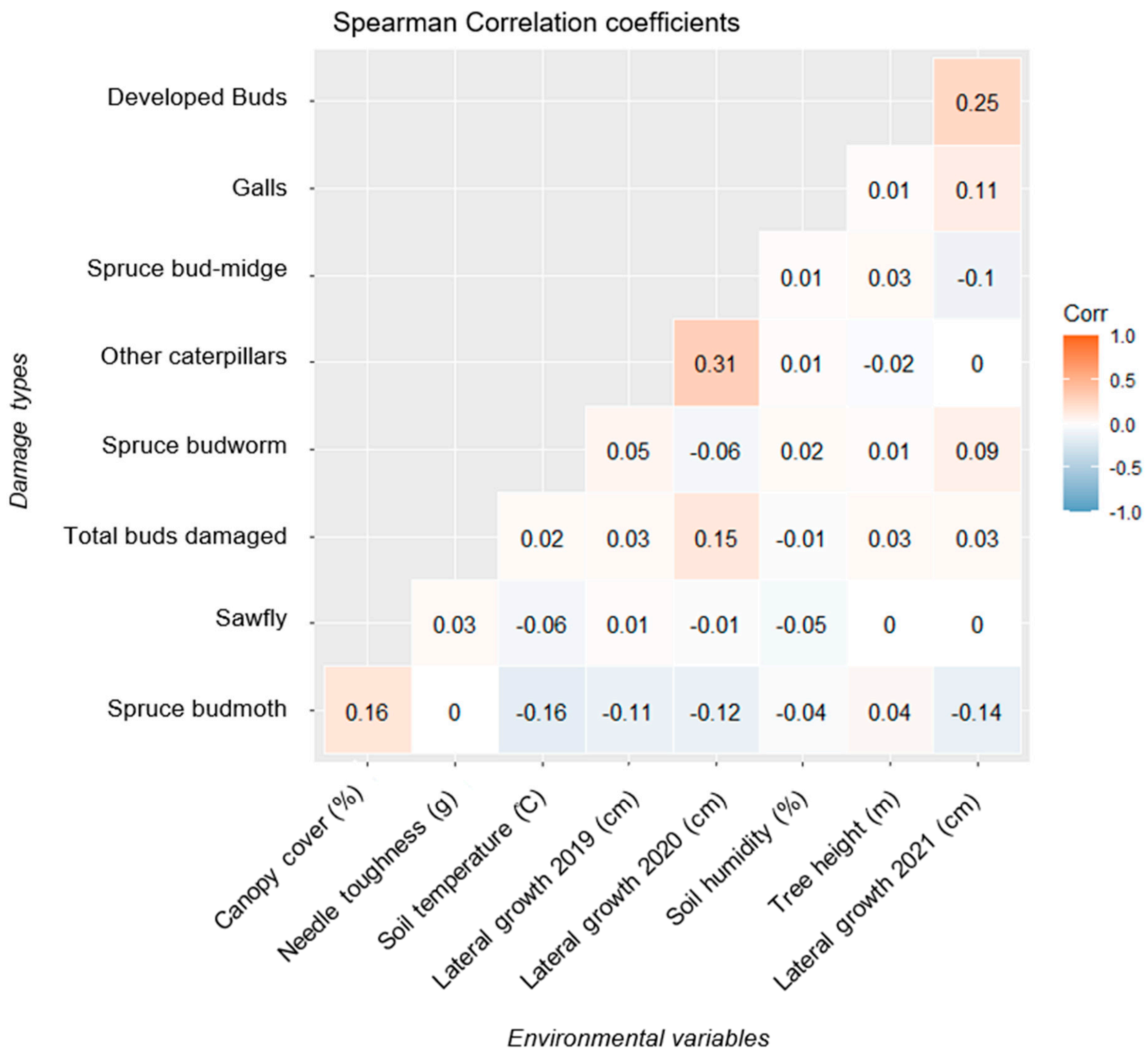

Figure S2. Spearman's correlation coefficients matrix of environmental variables and damage types was executed for branch data, where the correlation coefficient ranged from -1 to 1. A value close to 1 indicates a strong positive correlation, close to -1 indicates a strong negative correlation, and close to 0 indicates a weak or no correlation.
